# A global dataset of salmonid biomass in streams

**DOI:** 10.1038/s41597-024-04026-0

**Published:** 2024-10-29

**Authors:** Kyleisha J. Foote, James W. A. Grant, Pascale M. Biron

**Affiliations:** 1 Department of Geography, Planning and Environment, Concordia University, 1455 De Maisonneuve Blvd W., Montreal, H3G 1M8 Quebec Canada; 2 Department of Biology, Concordia University, 7141 Sherbrooke Street West, Montreal, H4B 1R6 Quebec Canada

**Keywords:** Freshwater ecology, Limnology

## Abstract

Salmonid fishes are arguably one of the most studied fish taxa on Earth, but little is known about their biomass range in many parts of the world. We created a dataset of estimated salmonid biomass using published material of over 1000 rivers, covering 27 countries and 11 species. The dataset, spanning 84 years of data, is the largest known compilation of published studies on salmonid biomass in streams, allowing detailed analyses of differences in biomass by species, region, period, and sampling techniques. Production is also recorded for 194 rivers, allowing further analyses and relationships between biomass and production to be explored. There is scope to expand the list of variables in the dataset, which would be useful to the scientific community as it would enable models to be developed to predict salmonid biomass and production, among many other analyses.

## Background & Summary

Undoubtedly, one of the most researched fish taxa on the planet is the salmonid family, largely because of their economic, social and cultural significance^1–3^. Salmonid biomass (g/m^2^) and production (g/m^2^/yr) have been estimated since at least the 1950s for many rivers around the world where they are both native and exotic^4,5^, but comprehensive compilations on the global range of salmonid biomass and production have not been completed for several decades (i.e. since Mann & Penczak in 1986^6^). Biomass and production estimates for stream salmonids can vary widely^7–9^, depending on species sampled, life-stage, the number of species present, fish sampling methods^10–12^, and many environmental conditions^13–15^. Attempts to predict salmonid biomass using models have typically been developed for a particular region^16–18^, but have been less useful when applied to other areas of the world (e.g.^14,19^). Additionally, many of the variables included in these models are difficult to quantify for a large global dataset without additional field sampling.

This data descriptor introduces a dataset of published salmonid biomass and production estimates from streams around the world, which can be found at FigShare^20^. It describes the methods used to create the dataset including the systematic review to collect the data. To synthesize the published global biomass estimates of stream salmonids, we conducted a systematic review focusing on the genera *Salmo*, *Oncorhynchus* and *Salvelinus*. PostgreSQL (version 14.13) was used to create a SQL (Structured Query Language) dataset to store biomass estimates, along with other study variables, density, and production (if it was reported). To our knowledge, this is the largest dataset of published biomass and production studies for stream salmonids.

Biomass estimates in the dataset span 84 years, from 1937 to 2021^20^. In total, 1063 rivers are included that contain data on salmonid biomass. For each biomass estimate, information is provided about its location, species, fish sampling method, river and site information and reference and project information is provided for each source in the dataset^20^. Production estimates are included for 194 rivers in the dataset.

The data are useful for exploring questions around biomass limitations and differences in stream salmonid biomass and production. Additionally, relationships between biomass and production can be analysed. Goals for collection of the data were to explore the range of biomass in stream salmonids, determine if previous estimated upper limits of biomass and production are valid, and test predictions about how key variables that are readily available for most rivers affect salmonid biomass in rivers^21^ (Table 1).

**Table 1:** Key variables included in the dataset and the type or method of measurement.

Variable	Measure
Species	Salmonid species
Number of species	Number of different salmonid species caught and reported in biomass estimate
Migratory status	Anadromous, resident or semi-anadromous (see Life history section below)
Species status	Native or exotic population, or mixture of both (if two species are caught and biomass is reported together)
Country and region	Country and region of sampling location
Location	Latitude and longitude (estimated if not provided)
Stream width	Mean, standard deviation, number of samples
Stream depth	Mean, standard deviation, number of samples
Elevation	Estimated if not provided, averaged for multiple sites
Fishing method	Method used to catch fish, e.g. electrofishing, snorkelling
Season	Season(s) fished.
Year of fishing	Last year of fish sampling if multiple years
Number of years of sampling	Number of years that were fished. Equals 1 if 1 or less.

## Methods

**Fig. 1:**
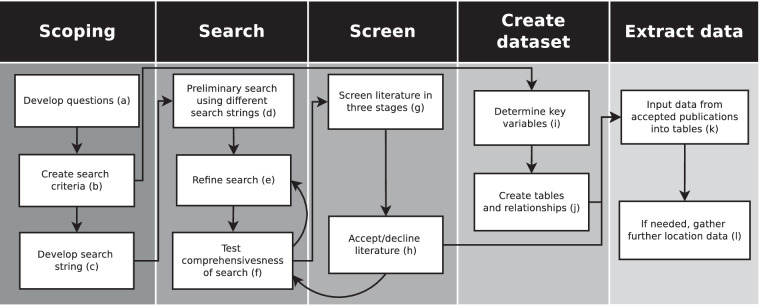
Overview of steps following to create and fill the dataset. Letters refer to further details described in the text.

An overview of the steps used to create the dataset is shown in Fig. 1. All the steps except creating the dataset are related to the systematic review, described below.

### Systematic review

To generate the dataset of published stream salmonid biomass^21^, a systematic review was conducted. Systematic review methodology^22^ was followed to obtain peer-reviewed articles and grey literature.

Key questions and priorities (Fig. 1a) for data collection were: (1) to determine the range of salmonid biomass and production in streams across the Earth; (2) to determine if previous estimated upper limits of biomass and production are valid; and (3) to test predictions on how key variables affect salmonid biomass in rivers (see^21^ for further information). Search criteria (Table 2) and search strings were developed to incorporate relevant studies from the literature (Fig. 1b,c). A Review Team conducted the search in English from two main sources: (1) Web of Science (WOS, www.webofscience.com) database using keywords; and (2) reference lists and cited data from included articles and known studies to capture papers published before 1979.

**Table 2:** Criteria for inclusion in the systematic search.

Field	Include	Exclude
Ecosystem	Rivers and streams, artificial channels that are connected to natural ones	Lakes, marine and coastal environment, lab experiments
Species	Salmonid genera *Salmo*, *Oncorhynchus* and *Salvelinus*	Non-salmonid species. Salmonid species from other genera. Estimates that include salmonids and non-salmonids
Location	Global	—
Abundance data	A measure of biomass per unit area (g/m^2^) – mean and variance if provided. Production (g/m^2^/yr) and density data (no./m^2^) recorded if reported	No quantitative data. If target fish were stocked as part of an experiment before sampling
Time period	Any time before 31 July 2023. Note that the Web of Science database starts from 1979 but earlier studies were included by searching the literature cited of papers included in the analysis	Studies published after 31 July 2023
Publication format	Published data in articles, reports, books, conference proceedings and theses	Online databases, unpublished surveys, data from presentations, personal communications

A preliminary search was conducted to examine the extent of literature using different combinations of search strings (Fig. 1d). During the initial search, the following keywords were used in a WOS search for the date range 1979 (start date of the WOS database) to 1 July 2021: TOPIC: (salmonid OR salmo* OR trout OR *Salvelinus* OR *Oncorhynchus*) AND TOPIC: (biomass OR abundance OR product* OR ‘standing stock’ OR densit*) AND TOPIC (river OR stream), where an asterisk (*) denotes a wildcard that can represent any collection of characters. This produced 7,564 results. The results were sorted by relevance and then the first 500 papers were screened (see Supplementary Table S1). The acceptance rate for biomass studies was 9.2%. Due to the large scope of studies that report density, it was excluded from our criteria and we decided that density would only be included in the dataset if biomass was also reported. Hence, the initial search string was modified (Fig. 1e). We focussed on biomass as the keyword rather than production because more studies measured biomass than production and most studies of production included a measure of biomass.

The refined search terms for the final WOS database search for the date range 1979 to 31 July 2023 were: (salmonid OR salmon OR trout OR *Salvelinus* OR *Oncorhynchus*) AND (biomass) AND (river OR stream), and resulted in a total of 1011 studies. The entire list of the final search was screened (Fig. 1g,h) for inclusion criteria in four stages: (i) title, (ii) abstract, (iii) partial article, and (iv) full text. Results at each stage of screening were recorded (Table S1).

To ensure a comprehensive inclusion of grey literature and older literature not included in the WOS search, reference lists from included articles were screened for possible inclusion. Furthermore, a Google Scholar (scholar.google.com/) search found additional studies that did not appear in the WOS search, because they were published before 1979 or were categorised as grey literature. With French expertise on the team, a basic search was also conducted in French (see Table S1). The comprehensiveness of the final search was tested by determining if benchmark articles appeared in the WOS database search (Fig. 1f; see Table S1).

**Fig. 2:**
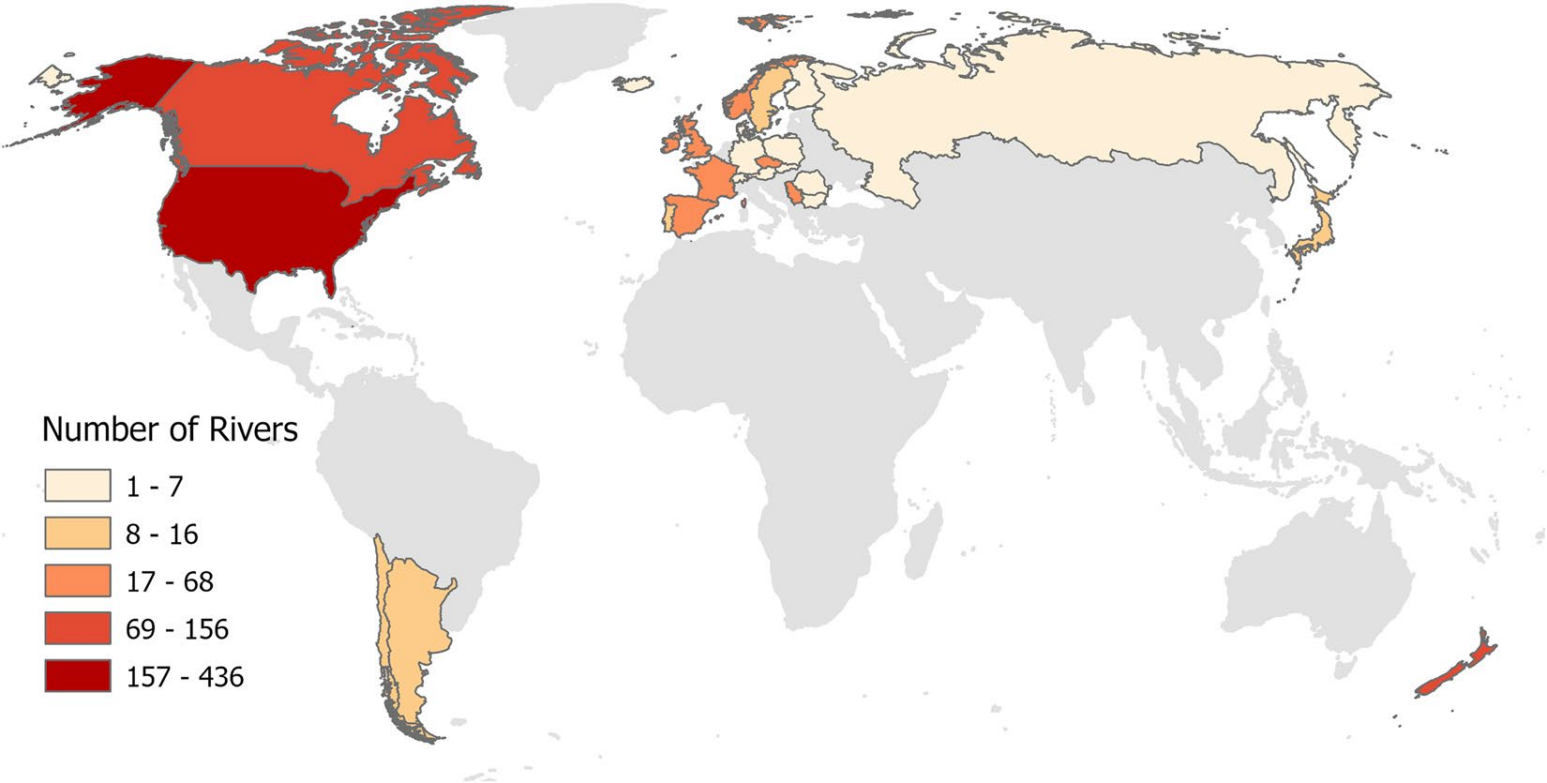
Number of rivers in each country included in the dataset. No data were available for countries in grey.

A total of 240 studies from 27 countries, on 1063 different rivers, were included in our dataset^20^ (Fig. 2). From the 1011 results in the WOS search, 146 met the acceptance criteria (14.4% acceptance rate). A further 94 publications were sourced from the reference lists of included studies, of which 35 were published before 1979 (the start year of the WOS database). The majority (87%) of the 240 studies included in the dataset were published as journal articles. Searches were mainly conducted in English (one search was carried out in French, see Table S1), but automated translators and fluent speakers were used where necessary for studies found in other languages. Only 4.1% of accepted publications were in languages other than English and included: French (5 studies), Czech (3), Slovak (1), and Spanish (1), while 2.1% (21) of publications in the total WOS search were not in English.

### Dataset creation

**Fig. 3:**
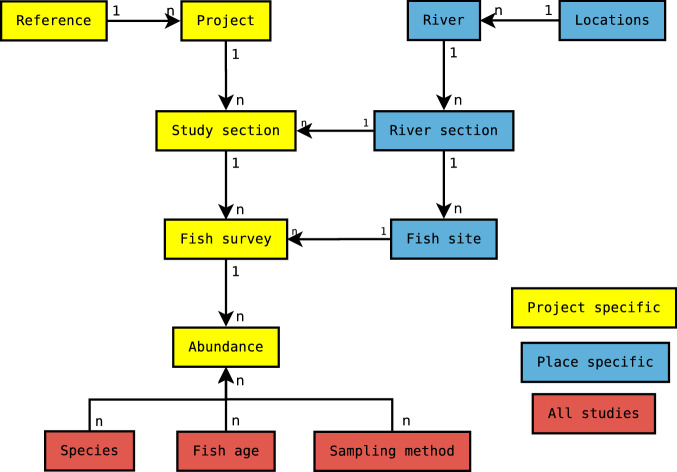
Dataset overview. Boxes represent separate tables and are related with a common ID by either a one-to-many (1-n) or many-to-many (n-n) relationship. Information in tables can be recorded for each project (yellow), for each place (blue), or relate to all studies (red). See Table S4 for the contents of each table and related IDs.

PostgreSQL was used to create a SQL (Structured Query Language) dataset to store biomass estimates, along with key study variables (Table 1; Fig. 1i) and other information (see Tables 3, 5), as well as production and density if it was reported. Twelve tables were created to store the data in a spreadsheet format and common ID numbers were used to relate them to each other (Figs. 1j, 3; Tables S2, S4).

Two types of relationships between tables existed in our dataset: one-to-many and many-to-many. The most common is the one-to-many relationship, where one record in a table is related to one or more records in another table. For example, one location id can have many rivers, but one river can only have one location id. The many-to-many relationship exists where multiple records in one table are related to multiple records in another table. For example, a fish survey site may have many species, and fishing methods recorded, and likewise, species and fishing methods can be recorded over many fish surveys. Tables can be specific to each project (records were related to only one project), place specific (the same place can be attached to many different projects), or can relate to all projects (Fig. 3). Projects are specific to each referenced publication. Most references have one project, but some have multiple projects. Projects are defined by different study objectives - i.e. one project might assess the effects of restoration, whereas another looks at long-term salmonid productivity; if reported in the same publication they will be classed as two different projects.

### Data input

The smallest spatial scale is the fish sampling site (fish site and fish survey; Fig. 3, Table S2). Estimated biomass was recorded in the dataset at the smallest reported scale by species (linked to fish survey). Multiple fishing sites that had similar conditions were combined into a river section and study section, measured at the same spatial scale, but the former can be shared across projects, whereas the later provides project specific details. If studies reported one biomass estimate over several rivers, it was only included in the dataset if environmental conditions were very similar among rivers and rivers were close together (i.e. in the same catchment); in most cases these studies were not included.

Most of the information on the variables were taken directly from the publication or supplementary publications (Fig. 1k). If sufficient detail was not given for locations of sites (e.g., latitude and longitude), these were estimated in Google Earth (www.google.ca/earth/) based on descriptions given (Fig. 1l). Hence, latitude and longitude may not be precise for many studies as this information was often omitted. Watershed names were found in Google Earth or using topographical maps if not provided in the study. Drainage area and length of rivers were found in a Google Search if not reported in the publications. All publications included in the dataset are referenced in the reference list of this paper^4,7–9,13,14,16,19,23–254^.

Initially, whether populations were stocked was recorded in the dataset. However, the reporting of stocking history was inconsistent between publications and many studies did not report this information. Due to this inconsistency, it was not deemed reliable to categorize a stream as stocked or not. We avoided studies where streams had been recently stocked with adult fish (if known) and where the effect of stocking was being tested.

### Life history

Migratory strategies for species were recorded in the species table^20^ (see Table S2), and included simplified categories of stream resident, anadromous, and anadromous and/or resident. Migratory status was recorded at the species level rather than the population level. While there is intraspecific variation in life history, those categorized as stream resident or anadromous were overwhelmingly represented in our data set. Thus, we separated species into general stream resident and anadromous species, with a mix being where two or more species are present belonging to both categories, or with species that are often semi-anadromous (i.e. cutthroat trout). Stream resident species included: brown trout (*Salmo trutta*) (50% of single species estimates in the dataset), brook trout (*Salvelinus fontinalis*), rainbow trout *(Oncorhynchus mykiss*), bull trout (*Salvelinus confluentus*), and some sub-species of cutthroat trout (*Oncorhynchus clarkii*). Anadromous populations included: Atlantic salmon (*Salmo salar*) steelhead trout (*Oncorhynchus mykiss*), Chinook salmon (*Oncorhynchus tshawytscha*), and coho salmon (*Oncorhynchus kisutch*). In the mixed category were: rainbow/steelhead (where no differentiation was given between them), cutthroat trout, masu salmon (*Oncorhynchus masou*), Arctic char (*Salvelinus alpinus*), and Dolly Varden trout (*Salvelinus malma*). The majority of brown trout populations were resident. However, there may be sea-run populations of brown trout included in the dataset but we avoided including adult biomass estimates where this was the case. No other salmonid species were included in the dataset.

### Interacting with the data

Data is provided as csv files with each table in Fig. 3 in a separate csv file^20^. The entire dataset is also provided in one csv file (dataset_salmon_full.csv). The data can then be used in the platform or language of choice (e.g. R, GIS, Python).

Separate tables can be joined using primary and foreign keys (see Table S3 for information on data types). Several joins are needed to join multiple tables. Biomass can be estimated at the fish survey scale, and can be summed for all salmonid species. To obtain an estimate at larger scales (river section and river) biomass is averaged (see Usage Notes). Biomass can then be assessed using variables in the dataset at the scale of interest. R code is provided with examples on joining tables and aggregating data using variables in the dataset^20^.

## Data Records

All data tables are deposited on FigShare^20^. Table S4 provides information on the structure of each table (csv file) in the dataset, including the data type, any constraints, and units for each column. Descriptions of data types and constraints included in the dataset are provided in Table S3. A full list of sources for the dataset are provided in a spreadsheet linked to references ids (ReferenceList.xlsx) as well as a pdf (ReferenceListDataset.pdf)^20^.

## Technical Validation

### Technical validation of search

Systematic review methodology^22^ was followed to ensure the search was comprehensive and biases were limited or accounted for (see Table S1 for full details).

An initial search to capture a large sum of publications (7564) was performed to review the scope of the literature. The first 500 ordered by relevance were searched for inclusion criteria. The overall acceptance rate was 9.2%, starting from 15% in the first 100 results and declining to 4% in the last 100 results (400–500). The final search (1011 results) was ordered by relevance and the acceptance rate for the first 100 was 26%, ending in 4% for the last 100 (average of 14.4%). The authors determined that 4% was an acceptable rate to end the search. Accepted articles from the initial search (benchmark articles) were tested for inclusion in the final WOS search with 100 per cent recovery. This indicates that continuing the search past the initial relevant 500 articles may not produce additional accepted publications.

Reference lists from accepted articles were screened for inclusion criteria, and those that met the criteria were then tested for inclusion in the final WOS search. To further ensure the robustness of the search, the first 100 results in a Google Scholar search (ordered by relevance) using the final search string were screened for inclusion criteria to capture publications that may not be included in the WOS search (grey literature and studies published before 1979).

To ensure the studies are not double counted, unique inputs are required for author(s) and river names in the dataset. For example, if a publication was entered a second time from the same author (e.g. Foote *et al*.), it would not be accepted and previous entries with the same author(s) name were checked for duplication. Where studies had been updated, only the most recent data were included or data were included in separate time periods (so study years do not overlap). If sampling years between studies were consecutive and there were few changes in the stream, an average was calculated over all years. If the publication was a different study, letters were used to distinguish between them (e.g. Foote *et al*. a; Foote *et al*. b). Likewise, river names could not be identical to ensure a river only had one River ID and to pick up duplicate data from different publications.

### Technical validation of data

Manual input of a large quantity of data is likely to result in some error. To ensure errors were minimal when inputting data, all variables were visualised as frequency histograms, scatter plots (qqPlot), and/or box plots in R (version 4.1.2). Outliers in these plots were checked for validity with the original publication and any errors were corrected. Random validation checks of publications were also carried out periodically to ensure data were entered correctly and at least 12 per cent of studies (30 out of 240) were carefully checked by two people.

## Usage Notes

Data in tables can be joined using common ID numbers. Refer to Table S4 for IDs across tables. Multiple joins may be needed to join several tables. Code for joining tables in R is available, using the merge function (see R document). NA values can be ignored in R when analysing (e.g.!is.na(*dataframe*), na.rm = T, na.omit(*dataframe*)).

To calculate average biomass at the river scale, data will first need to be summed at the fish survey scale. Each fish survey id represents a length of stream that has been fished at that site in the period of time outlined in the fish survey table. Multiple salmonid species and age classes caught at the same time will share a fish survey id, but may have different abundance ids if they are reported separately in publications. By summing at the fish survey id scale, an overall salmonid biomass estimate (or other abundance estimate) can be obtained.

To do this, data should be summed using the fish survey id as the aggregate factor, as that is the level at which fish passes occurred and where biomass data was provided by the literature. Data can be separated by species, age class, or other variables at this stage, or it can be summed for all variables (so biomass of all salmonid species fished at a site would be combined). The aggregate function in R can be used to sum data at this level (examples provided in R code).

The summed data can then be averaged by river section id or river id. By first summing the data we can ensure that data at each survey incident is not counted as separate fishing occurrences, and thus biomass for that stream section would be lower. Other variables can also be considered at this stage.

We have provided examples in the R code on combining sub-species and multiple species with one biomass estimate (as One species or Two species). At present in the dataset whether populations are exotic or native are coded as true or false, hence, we provide an example to rename these to ‘Exotic’, ‘Native’ or ‘Both’.

### Integrating other datasets

Spatial joins can be used in GIS to connect with other spatial datasets (e.g. based on latitude and longitude details using a buffer around the points if needed). We have not attempted to merge the dataset with other data in R, but users of the dataset may wish to explore this option.

## Supplementary information

Supplementary Material Foote et al.

## Data Availability

The dataset was generated by inputting variables from published material. All the variables we entered are listed in Table S2. Data outputs are available as csv files for ease of re-use. As such there was no custom code to generate the data. Code for joining data tables and interacting with the data in R is available in FigShare.
