# Supplementary material for: A global dataset of salmonid biomass in streams
Source: Sci Data. 2024 Oct 29;11:1172. doi: 10.1038/s41597-024-04026-0 (PMC11522555; doi:10.1038/s41597-024-04026-0)
Supplement: Supplementary file 1 — Supplementary Material Foote et al. [file 41597_2024_4026_MOESM1_ESM.pdf]

Supplementary Material

A global dataset of salmonid biomass in streams

Kyleisha J. Foote[1]*, James W.A. Grant[2], Pascale M. Biron[1]

[1]Department of Geography, Planning and Environment, Concordia University, 1455 De Maisonneuve Blvd W., Montreal, Quebec, Canada, H3G 1M8,

Email: K. J. Foote*: kyleisha.foote@mail.concordia.ca

P. M. Biron: pascale.biron@concordia.ca

[2]Department of Biology, Concordia University, 7141 Sherbrooke Street West, Montreal, Quebec H4B 1R6, Canada. Email: james.grant@concordia.ca

*Corresponding author: kyleisha.foote@mail.concordia.ca

Table of Contents

Table S1. Our systematic review methodology, following some of the guidelines from the Collaboration for Environmental Evidence (2018)..3

Table S2. Descriptions of tables in the dataset and data extracted if reported..........................9

Table S3. Descriptions on data types used in the tables..11

Table S4. Descriptions of each column and data type in tables. Table name and brief description is in italics before the table columns..12

Table S1. *Our systematic review methodology, following some of the guidelines from the Collaboration for Environmental Evidence (2018)*

Steps in systematic review	Our methods
**1. Conducting a search**	
Including peer-reviewed and grey literature	Our analysis included 208 (87%) journal articles and 32 (13%) grey literature items (reports, theses, book chapters, and conference proceedings).
Test comprehensiveness of search	All of the items that met the acceptance criteria (Table 2 in main text) in the initial search (most relevant 500 of 7,564 studies (benchmark articles)) were present in the final Web of Science (WOS) search. Out of the total 240 publications in the database, 146 were sourced in the WOS search. For items not included in the WOS search (94 total publications), 33 were published before 1979 (the start date of the WOS database), 2 were published in 1979 (may have missed being included in the WOS database), 24 were grey literature, 19 were focused on production or growth rather than biomass (but still reported biomass), 7 were restoration studies that may not have mentioned biomass in the abstract, 2 were not in English (so would be less likely to appear in an English search), and the remaining 7 were published in journals that may not be included in the WOS database.
All search terms provided	During the initial search, the following keywords were used in the Web of Science search for the date range 1979 to 1 July 2021: TOPIC: (salmonid OR salmon OR trout OR *salvelinus* OR *oncorhynchus*) AND TOPIC: (biomass OR abundance OR product* OR 'standing stock') AND TOPIC (river OR stream), where an asterisk (*) denotes a wildcard that can represent any collection of characters.<br>Search terms were updated to: (salmonid OR salmon OR trout OR *salvelinus* OR *Oncorhynchus*) AND (biomass) AND (river OR stream).
Databases, search engines and search	The only database that a comprehensive search was conducted was the Web of

dates reported	Science. Keywords were also searched in Google Scholar for grey literature and publications before 1979 in both English and French. The initial WOS search was conducted in July 2021 with the updated search terms used in December 2021 and repeated in August 2023.
Updates to searches	An updated search was conducted in August 2023 to capture recently published articles.
Description of grey literature searches reported	Reference lists of review articles and accepted articles that mentioned biomass studies were searched. Salmonid researchers and experts were contacted to find published reports and hard to find literature. Google Scholar was used to capture additional grey literature.<br>Of the 32 grey literature items included, 14 were reports, 12 proceedings (mostly from conferences), 2 book chapters, 1 book, 2 special publications, and 1 thesis.
Limitations	Search strings in WOS were only conducted in English, however, publications that were in other languages were scanned and included if they met the acceptance criteria data. A simplified search was conducted in French in Google Scholar.<br>The database only included studies that were reported, or could be converted to g/m^2. Many studies reported biomass in weight only or by linear stream length and did not include width measures to convert it to an area measurement.<br>Many studies that focused on production also reported biomass but did not get picked up using the key words. Due to time constraints, a further search was not conducted.<br>Publications and the number of rivers were overwhelmingly focussed in three countries (United States, Canada, and New Zealand), which could indicate an English language bias. In order to determine if data from each country was in proportion to its land area, we looked at the ratio of the proportion of rivers represented from each country in the dataset over the proportion of that country's area in the dataset. This was calculated as follows:

	Proportion of land area for country (area) = land area of country / total land area of all countries in the dataset * 100. <br>Proportion of rivers for country$_i$ (rivers) = number of rivers with data from country / total number of rivers in the data set *100. <br>The ratio is then calculated as: area/rivers. <br>This showed that some countries in Europe (as well as New Zealand and the UK) are over-represented for their land area (ratio >10: Czech Republic, Ireland, Liechtenstein, and Serbia), while the US and Canada have a ratio of 0.67 and 1.89, respectively. <br>Focus on high biomass rivers: Published material is focused on sites with a biomass higher than zero – so zeros are generally not counted. There may be a bias to study and publish about high productivity streams.
**2. Eligibility screening**	
Eligibility criteria defined	See Table 2 in main text
Stages of screening literature	Search results were screened in four stages: (*i*) title - the titles were first scanned for eligibility; (*ii*) abstract - abstracts or summaries of those that seemed relevant or were unclear were scanned for eligibility criteria; (iii) partial article – the text was scanned for inclusion criteria but if deemed very irrelevant the whole text was not scanned and (*iv*) full text - if deemed relevant or it was unclear, the full text was searched for inclusion criteria. References from included articles were screened in these stages.
Eligibility discussed among authors	Due to the large number of search results in the initial search where density was included, it was decided that only biomass would be included as a criterion. Density and production would only be recorded if the publication reported biomass. Salmonid species were narrowed down to three main genera due to most of the literature focused on these genera. <br>Studies that were assessing the effects of fish stocking were not included, as the results could be short lived, but streams that had been stocked in the past were included, as it

	assumed that populations had reached a stable state, and it was not always reported if stocking had taken place or not.<br>Spawning anadromous fish were not included due to most of the growth being undertaken at sea.<br>Only published data were included to limit the scope of the study and exclude data that may not have been rigorously sampled.
Numbers of articles found in search and number excluded	Number of results from Web of Science search: Initial (7,564) – sorted by relevance and first 500 were scanned. Updated search terms December 2021 (964), then July 2023 (47).<br>From the updated search (1011 publications), 756 were scanned at stage *iv*, 237 at *iii*, 10 at *ii*, and 8 at *i*.<br>Due to the large amount of extra material searched (not in the WOS search) and overlapping of studies, the total number of publications searched and excluded were not counted.
Reasons for exclusion	The main reasons for publications that reported biomass being excluded were: biomass not reported in the right units (and no way to convert it), only lakes or coastal areas were surveyed, biomass was for spawning anadromous fish, or fish were stocked directly before sampling (so the effect of stocked was being tested).
List of studies included in the meta-analysis	See the reference list of the main paper and the DataReferenceList pdf in FigShare.

3. Data coding and data extraction

Data extracted	See Table S2 for data that were extracted from each study (if reported). If latitude and longitude were not given by the study authors, they were estimated based on study descriptions. There may be some inaccuracy here as exact locations were not always given.

Scale reported	Data were extracted and reported at multiple scales. Biomass was recorded at the fish survey scale for all studies, even if the biomass reported was averaged over many sites (these were classed as the fish survey scale in our database). Data can then be averaged for reaches, river sections or whole rivers (see methodology section of main text for more details).
**4. Critical appraisal of study validity**	
Identify sources of bias and correct or perform sensitivity analysis	Publication bias (rivers that have high known biomass and production are more likely to be studied and reported on). Attempts were made to include grey literature, which may include rivers with lower biomass – many of these encompassed surveys of a large area. Our search and acceptance criteria were not only focused on high productivity, and included studies that may have productivity limitations and restoration studies that often focus on low productivity/biomass streams.<br><br>Language bias (major search was conducted in English, thus will result in English speaking countries being overly represented). We searched publications in other languages that were captured in the WOS search (21 in total). Many of the search terms will also be applicable in other languages.<br><br>Given the French expertise on the team, we also conducted a simplified search in French in Google Scholar using the following terms (salmonid OR salmo* OR truite OR Salvelinus OR Oncorhynchus) AND (biomasse) AND (fleuve OR ruisseau OR rivière OR “cours d’eau”).<br><br>Availability bias (only easily available studies are included) – to counteract this we searched reference lists of included studies to obtain literature that may not appear in database searches, and obtained some grey literature from salmonid researchers.<br><br>Duplication bias (studies may be published more than once) – studies were checked when included and duplicates were removed. Where studies had been updated, only the most recent data were included or data were included in separate time periods (so study years do not overlap). To ensure the same publication was not entered into the database more than once, references names could not be identical – if a publication was entered a second time from the same author (e.g. Foote et al.), it would not be

	accepted and previous entries with the same author name were checked. If the publication was a different study, letters were used to distinguish between them (e.g. Foote et al. a; Foote et al. b). Likewise, river names could not be identical to ensure a river only had one River ID and to pick up duplicated data from different publications.
**5. Data synthesis**	
Information on eligible studies	Full bibliographic information is provided in the reference list of the main paper and the DataReferenceList pdf in FigShare.

Table S2. *Descriptions of tables in the dataset and data extracted if reported.*

Table name	Description	Items included
Reference	Reference information and identification for each study	Reference id, author, publication year, publication type (e.g. article, report, book chapter, conference proceeding), other references that include the same rivers or have additional information
Project	Project details for each study. Most references have one project, but some have multiple projects.	Project id, reference id, project start and end date, number of years the project spans, number of streams/rivers that have biomass measures, project type (i.e. population study, restoration study)
Location	General location details for the study. Shared over studies.	Location id, geographical region, province (or state or geopolitical region), country, continent
River	River, stream or creek sampled for fish. Can be shared over different studies.	River id, location id, river name, drainage area (or whole river), river length, watershed name (name of basin that river drains into)
River section	Section of the river studied. Each study may have several river sections where obvious changes occurred. Can be shared over different studies.	River section id, river id, latitude, longitude
Study section	Section of the river that is specific to the study. Same location as river section. Study sections are not shared among studies.	Study section id, river section id, project id, study length, number of treatments (if restoration or intended modifications occurred at the site)

Fish site	Site where fish sampling occurred. Can be shared over different studies.	Site id, river section id, site name, stream order, elevation
Fish survey	Site details where fish sampling occurred, specific to each study.	Fish survey id, site id, study section id, length fished, sections fished, mean width and depth, start and end dates of fishing, number of years that were fished (1 if 1 or less), seasons fished
Species	Species details	Species id, common name, scientific name, migratory strategy (resident, anadromous, or semi-anadromous (mixture of both or a species that is frequently both))
Sampling method	Method of fish sampling for the study for all studies.	Fishing id, fishing type, number of removals or passes, fishing methods, reference (for sampling method)
Fish age	Fish age classes	Age id, age class, age category
Abundance	Details about fish catch, specific to each study. Separated by species and age class if reported separately in the study.	Abundance id, fish survey id, species id, age id, fishing id, number of species included in the measure, biomass, density and production (mean, standard deviation (SD) and sample size (N) for all), exotic (true or false).

Table S3. *Descriptions on data types used in the tables.*

Data type	Description
Serial	Creates unique identifier columns. An integer column is created and default values are assigned from a sequence generator
Varchar(size)	A variable length string (can contain letters, numbers, and special characters). The size parameter specifies the maximum string length.
Smallint	A small integer. Range is from -32768 to +32767
Integer or int	A medium Integer. Range is from -2147483648 to +2147483647
Real	Variable precision, inexact. Range is at least 6 decimal digits.
Numeric	Can store numbers with a very large number of digits. Exact numbers. Range is up to 131072 digits before the decimal point; up to 16383 digits after the decimal point
Date	Date columns are input in the following format: YYYY-MM-DD
Constraints	
Primary key	Indicates that a column can be used as a unique identifier for rows in the table. Requires that the values be both unique and not null
Foreign key	Specifies that the values in a column must match the values appearing in some row of another table. This maintains the *referential integrity* between two related tables.
Unique	Ensure that the data contained in a column is unique among all the rows in the table
Not null	A column must not assume the null value

Table S4. *Descriptions of each column and data type in tables. Table name and brief description is in italics before the table columns.*

**Column name**	**Column description**	**Data type and constraints[a], units**
Reference table – details about the reference		
reference_id	Unique id for reference	Serial, primary key
author	First author last name. Both names listed if two authors. For three or more authors et al. is used after the first author	Varchar, unique, not null
pub_date	Year of publication	Smallint, YYYY
pub_type	Type of publication, e.g. article, chapter, report	Varchar(50)
other_references	Lists other references that might have the same data, are the same project, or the same river	Varchar(500)
Project table – details about each project		
project_id	Unique id for project	Serial, primary key
reference_id	Unique id from reference table	Foreign key – refers to reference table
proj_start_year	Start year of whole project	Smallint, YYYY
proj_end_year	End year of whole project	Smallint, YYYY
proj_num_years	Number of years that project spans	Integer, 1 for projects 1 year or less
num_streams	Number of different streams fished	Smallint
num_species	Number of salmonid species caught	Smallint
proj_type	Main focus of project – e.g. population study, restoration	Varchar(50)
proj_notes	Other details about project focus	Varchar
Locations table – general information about regions in database		
location_id	Unique id for location	Serial, primary key

region	Unique name for geographical region	Varchar(250), unique
province	Province, state or region name	Varchar(25)
province_abbr	Abbreviation for province or state	Varchar(10)
country	Country name	Varchar(50), not null
continent	Continent name	Varchar(50)
River table – information about each river in database		
river_id	Unique id for river	Serial, primary key
location_id	Unique id from location table	Foreign key – refers to location table
river_name	Unique name for river – two rivers cannot have the same name	Varchar(50), not null, unique
drainage_area	Drainage area of whole river	Numeric, km^2
river_length	Length of whole river	Numeric, km
watershed_name	Major watershed name that river drains into. If river drains into sea or major lake then watershed_name is the same as river	Varchar(50)
River section table – information about section of river in database		
river_section_id	Unique id for river section – section on river that is similar in terms of habitat characteristics or treatment characteristics	Serial, primary key
river_id	Unique id from river table	Foreign key – refers to river table, not null
lat	Latitude referring to river section, may be estimated or approximate as often not given	Real, decimal degrees
long	Longitude referring to river section, may be estimated or approximate as often not given	Real, decimal degrees
Study section table – information about section of river in each project		

study_section_id	Unique id for study section	Serial, primary key
river_section_id	Unique id from river section table	Foreign key – refers to river section table, not null
project_id	Unique id from project table	Foreign key – refers to project table, not null
study_length	Total length of study – combined length of all sites in the section	Numeric, meters
num_treatments	Number of different treatments if modifying stream conditions, e.g. restoration studies	Integer
Fish site table – site in database that is fished		
site_id	Unique id for fish site	Serial, primary key
river_section_id	Unique id from river section table	Foreign key – refers to river section table, not null
site_name	Name of site – attempting to correspond with names given in publication	Varchar(100)
stream_order	Stream order, taken from publication so not clear what definition is used. Width or upstream drainage area may be more useful for describing size of stream	Numeric
Fish survey table – information about each site that is fished, unique to a project		
fish_survey_id	Unique id for fish survey	Serial, primary key
study_section_id	Unique id from study section table	Foreign key – refers to study section table, not null
site_id	Unique id from fish site table	Foreign key – refers to fish site table
length_fished	Length of section fished – if reported biomass is combined for several sections then it is the total length for estimate	Numeric, meters

sections_fished	Number of sections for biomass estimate	Integer
width_mean	Width of site fished, or average if several sites. Generally wetted width was extracted but often no indication on the type of width measurement was given	Numeric, meters
width_sd	Standard deviation of width if several measurements given	Numeric
depth_mean	Depth of site fished, or average if several sites	Numeric, meters
depth_sd	Standard deviation of depth if several measurements given	Numeric
start_fish_date	First date of fishing	Date, YYYY-MM-DD
end_fish_date	Last date of fishing	Date, YYYY-MM-DD
num_years	Total number of years that were fished, not the number of years between start and end, 1 if 1 or less.	Numeric
seasons_fish	Seasons that were fished, combined if 3 seasons, annual if 4 seasons, unknown if not given	Varchar(25)
Species table – information about each species in the database		
species_id	Unique id for species	Serial, primary key
common_name	Common name of species, species are combined if reported together and can't separate estimates into individual species	Varchar, not null, unique
sciname	Scientific name of species	Varchar, not null
migstra	General migratory strategy – anadromous, resident, semi-anadromous or anadromous and/or resident	Varchar
num_species	Number of species in the species id	Integer

Fish age table – information about fish age classes in database		
age_id	Unique id for fish age	Serial, primary key
age_class	Age class of fish	Varchar, not null, unique
age_category	Broad category of age class – e.g. juvenile, adult, population. Classed as population if age isn't specified.	Varchar(25), not null
Fishing table – information about method of fishing		
fishing_id	Unique id for fishing method	Serial, primary key
fishing_type	General type of fishing – e.g. electrofishing, snorkeling	Varchar(100), not null
num_removals	Number of fish removals from the section	Integer
fishing_method	Description of fishing method	Varchar(500)
reference	Reference of fishing method if particular for study or need more details	Varchar(100)
Abundance table – information on fish catch		
abundance_id	Unique id for abundance estimate	Serial, primary key
fish_survey_id	Unique id from fish survey table	Foreign key – refers to fish survey table, not null
species_id	Unique id from species table	Foreign key – refers to species table, not null
age_id	Unique id from fish_age table	Foreign key – refers to fish_age table, not null
fishing_id	Unique id from fishing table	Foreign key – refers to fishing table, not null
num_species	Number of salmonid species included in estimate	Integer
biomass_mean	Mean biomass	Numeric, g/m^2
biomass_sd	Standard deviation of biomass	Numeric, Standard deviation g/m^2
nbiomass	Number of samples that estimate is based on	Integer

density_mean	Mean density	Numeric, num/m^2
density_sd	Standard deviation of density	Numeric, Standard deviation num/m^2
ndensity	Number of samples that density estimate is based on	Integer
production_mean	Mean production	Numeric, g/m^2/year
production_sd	Standard deviation of production	Numeric, Standard deviation g/m^2/yr
nproduction	Number of samples that production estimate is based on	Integer
exotic	Whether estimate is for exotic (true), native (false) or both (both) populations	Varchar(50), True, false, both

Notes: [a]Refer to Table S3 for data type and constraint descriptions.
